# Reproducibility of functional aortic analysis using MRI: the multi-ethnic study of atherosclerosis

**DOI:** 10.1186/1532-429X-17-S1-O67

**Published:** 2015-02-03

**Authors:** Chikara Noda, Bharath Ambale Venkatesh, Yoshiaki Ohyama, Chia-Ying Liu, Elzbieta H Chamera, Alban Redheuil, Gisela Teixido-Tura, Atul Chugh, Colin Wu, Gregory Hundley, David Bluemke, Joao A Lima

**Affiliations:** 1 Johns Hopkins University, Baltimore, MD, USA; 2 National Institutes of Health, Bethesda, MD, USA; 3 Groupe Hospitalier La Pitié Salpêtrière Sorbonne Universités, Paris, France; 4 Vall d'Hebron Hospital, Barcelona, Spain; 5 Jewish Hospital, Luisville, KY, USA; 6 Wake Forest University Health Sciences, Winston-salem, NC, USA

## Background

To assess the test-retest, intra- and inter-reader reliability of thoracic aorta measurements by magnetic resonance image (MRI).

## Methods

Twenty-five participants underwent aortic MRI twice over 13 ± 7 days. All aortic variables (ascending aortic area, descending aortic area, ascending and descending aortic strain [maximum area minus minimum area divided by minimum area], aortic arch transit time and pulse wave velocity [PWV]) from baseline and repeat MR were analyzed using a semi-automated method by the ARTFUN software. Phase contrast (PC) cine at the level of the pulmonary artery bifurcation was used for assessment of aortic area, aortic strain, and PWV. Steady State Free Precession (SSFP) sagittal images were used to measure aortic arch transit distance. To assess the inter-study reproducibility of aortic variables, we calculated intraclass correlation coefficient (ICC) for individual aortic measurements. Intra- and inter-observer variability was also assessed using the baseline MR data.

## Results

The mean age of these Twenty-five participants was 66 ± 7 years (18 males and 7 females). Of these, 28% had diabetes mellitus, 56% were hypertensive, 64% were current smokers, and 16% had hyperlipidemia. Mean ascending aortic strain had moderate inter-study reproducibility (11.53 ± 6.44 vs. 10.55 ± 6.64, p = 0.443, ICC = 0.53, p < 0.01). Mean descending aortic strain and PWV had good inter-study reproducibility (descending aortic strain: 8.65 ± 5.30 vs. 8.35 ± 5.26, p = 0.706, ICC = 0.74, p < 0.001; PVW: 9.92 ± 4.18 vs. 9.94 ± 4.55, p = 0.968, ICC = 0.77, p < 0.001, respectively). All aortic variables had excellent intra- and inter-observer reproducibility (intra-observer: ICC range, 0.87 to 0.99, inter-observer: ICC range, 0.56 to 0.99, respectively).

## Conclusions

Inter-study reproducibility of all aortic variables was acceptable. It was especially excellent in aortic area measurement. Intra-observer and inter-observer reproducibility of all aortic variables were also excellent. MRI can provide a repeatable method of measuring aortic functional parameters.

## Funding

This study was supported by contracts N01-HC-95159 through N01-HC-95169 from the National Heart, Lung, and Blood Institute and by grants UL1-RR-024156 and UL1-RR-025005 from NCRR.

**Table 1:** Inter-study reproducibility of Aortic function from MRI (n=25)

Aortic parameters	Exam1	Exam2	P value^a^	Mean difference	ICC
Max. Asc. Aortic area (cm^2^)	10.53 ± 1.91	10.61 ± 1.89	0.663	-0.07 ± 0.83	0.91**

Min. Asc. Aortic area (cm^2^)	9.47 ± 1.72	9.59 ± 1.48	0.452	-0.12 ± 0.77	0.89**

Max. Desc. Aortic area (cm^2^)	5.56 ± 1.24	5.57 ± 1.25	0.911	-0.01 ± 0.25	0.98**

Min. Desc. Aortic area (cm^2^)	5.14 ± 1.19	5.14 ± 1.16	0.891	-0.01 ± 0.26	0.98**

Transit time (ms)	16.52 ± 5.00	16.80 ± 5.44	0.721	-0.28 ± 3.87	0.73**

Transit distance (mm)	148.25 ± 27.59	146.90 ± 26.44	0.827	1.34 ± 30.43	0.38*

Asc. Aortic Strain (%)	11.53 ± 6.44	10.55 ± 6.64	0.443	0.99 ± 6.32	0.53*

Desc. Aortic Strain (%)	8.65 ± 5.30	8.35 ± 5.26	0.706	0.30 ± 3.90	0.74**

Pulse Wave Velocity (m/s)	9.92 ± 4.18	9.94 ± 4.55	0.968	-0.02 ± 3.03	0.77**

"Values are Mean ± SD, SD = standard deviation, ICC = Intraclass correlation coefficient. Max. = maximum, Min. = minimum, Asc. = ascending, Desc. = descending.

a = Paired t-test, ** = p <0.001, * = p <0.05."

